# Anti-nephrin autoantibodies in post-transplant recurrent focal segmental glomerulosclerosis: diagnostic advances and future directions

**DOI:** 10.1007/s10157-026-02830-z

**Published:** 2026-02-26

**Authors:** Yoko Shirai, Motoshi Hattori

**Affiliations:** 1 Department of Pediatric Nephrology, Tokyo Women’s Medical University, 8-1, Kawada-cho, Shinjuku-ku, Tokyo, 162-8666 Japan; 2 Kidney Disease Research Institute, Tokiwa Foundation, Jyoban Hospital, Iwaki, Fukushima Japan

**Keywords:** Nephrin, Slit diaphragm, Autoantibody, Focal segmental glomerulosclerosis, Transplantation

## Abstract

Nephrotic syndrome is a common kidney disease during childhood that is characterized by alterations in the glomerular filtration barrier and leads to protein loss in the urine. Approximately 90% of cases are classified as idiopathic nephrotic syndrome, most of which are histologically diagnosed as minimal change disease (MCD). Although the majority of patients achieve remission with steroid therapy, a subset develops steroid resistance and progresses to focal segmental glomerulosclerosis (FSGS) and kidney failure. Increasing evidence suggests that MCD and idiopathic FSGS represent a disease continuum, with FSGS reflecting a more advanced stage. Although several candidates have been proposed as circulating factors, none fully explains the disease pathogenesis. This landscape changed in 2022 with the discovery of anti-nephrin autoantibodies in MCD. Subsequently, we reported that circulating anti-nephrin autoantibodies were identified by ELISA in patients with post-transplant recurrent FSGS, and punctate IgG deposition colocalizing with nephrin was consistently detected in allograft biopsy specimens obtained during recurrence. Notably, these IgG deposits resolved following remission. Collectively, these findings suggest diffuse podocytopathies as autoantibody-mediated disorders and support a shift toward autoantibody-based disease classification. Experimental and clinical studies demonstrate that anti-nephrin autoantibodies induce nephrin phosphorylation. This process may be associated with nephrin endocytosis and subsequent cytoskeletal alterations. Additionally, autoantibodies targeting slit diaphragm molecules other than nephrin have been identified. However, the pathogenic roles of these autoantibodies remain to be clarified. Collectively, these findings highlight a complex, autoantibody-driven mechanism in diffuse podocytopathies and underscore the need for standardized assays and biomarker-driven classification strategies.

## Overview of diffuse podocytopathies

Pediatric nephrotic syndrome is the most common chronic kidney disease in childhood. In Japan, the annual incidence is approximately 6.5 per 100,000 children, which is three-to-four times higher than that observed in white populations, resulting in nearly 1000 newly diagnosed pediatric cases each year [1]. Approximately 90% of cases are classified as idiopathic nephrotic syndrome, of which nearly 80% are histologically diagnosed as minimal change disease (MCD) [2]. Most children with idiopathic nephrotic syndrome (80–90%) achieve complete remission with steroid therapy and are classified as having steroid-sensitive nephrotic syndrome. However, 10–20% of patients exhibit persistent proteinuria despite steroid treatment and are classified as steroid-resistant nephrotic syndrome [3]. While kidney prognosis is generally favorable in patients who achieve remission, those with persistent nephrotic syndrome frequently demonstrate focal segmental glomerulosclerosis (FSGS) on histological examination and progress to kidney failure [4]. Although MCD and FSGS were originally described as distinct entities because of differences in clinical presentation, pathological features, and prognosis, robust evidence supporting a clear separation is limited. In patients with steroid-resistant nephrotic syndrome who had been initially diagnosed as MCD on light microscopy but later progressed to FSGS, some researchers have suggested that an initial diagnosis of FSGS could have been missed owing to the focal and segmental nature of the FSGS lesion [5, 6]. Recently, idiopathic FSGS can be regarded as a more advanced phase of disease progression, in which responsiveness to therapy is reduced compared with earlier disease stages [7]. In recent years, the concept of diffuse podocytopathies has been proposed, integrating MCD and idiopathic FSGS as a unified disease spectrum [8].

## Historical perspectives on the pathogenesis of idiopathic nephrotic syndrome

The etiology of idiopathic nephrotic syndrome has long remained elusive. The efficacy of corticosteroids and calcineurin inhibitors, as well as reports of remission following measles or malaria infection, led to the hypothesis that T-cell dysfunction plays a central role [9]. Conversely, the effectiveness of plasmapheresis in post-transplant recurrent nephrotic syndrome suggested the involvement of circulating humoral factors [10]. The existence of circulating factors is supported by clinical observations showing that immediate post-transplant recurrence can be reversed by removal of the affected allograft and reimplantation into another recipient [11], as well as by experimental animal models in which serum from patients with recurrent focal segmental glomerulosclerosis induces proteinuria and podocyte foot process effacement [12]. Despite these observations, the precise identity of the circulating factors remains elusive. Proposed candidates include hemopexin (HPX), a protease [13, 14], cardiotrophin-like cytokine factor 1 (CLCF1), a member of the interleukin-6 cytokine family, and calcium/calmodulin-dependent serine protein kinase (CASK) [15, 16]. Elevated levels of soluble urokinase plasminogen activator receptor (suPAR), which is expressed by immune cells, endothelial cells, and podocytes, have been reported in various conditions associated with kidney injury and are capable of inducing podocyte foot process effacement [17]. However, administration of suPAR alone is insufficient to induce proteinuria in experimental models [18].

## Discovery of anti-nephrin autoantibodies

A monoclonal antibody targeting the B-cell surface antigen CD20, rituximab, has been shown to be effective in steroid-dependent nephrotic syndrome, suggesting that B cells are likely to be involved in the onset and relapse of nephrotic syndrome. Although the relapsing or steroid-dependent clinical course of MCD was not inconsistent with the possible involvement of autoantibodies, this concept conflicted with the prevailing view that IgG deposition is absent in the glomeruli of patients with MCD. While some reports described diffuse IgG staining on podocytes in MCD, IgG deposition was generally regarded as a hallmark of membranous nephropathy. Moreover, because no electron-dense deposits are observed on electron microscopy, such IgG staining was traditionally interpreted as nonspecific protein absorption by podocytes rather than true immune complex deposition [19]. However, studies using animal models had already demonstrated that antibodies targeting nephrin, a key structural component of the slit diaphragm, can induce massive proteinuria [20]. In addition, it had been recognized that kidney transplantation in patients with congenital nephrotic syndrome caused by mutations in the *NPHS1* gene, which encodes nephrin, can lead to the development of post-transplant nephrotic syndrome mediated by anti-nephrin antibodies in a subset of cases [21]. The disruption of cell–cell adhesion complexes by autoantibodies has also been described in other diseases, most notably pemphigus. In pemphigus, autoantibodies target desmogleins, which are critical cross-linking molecules within cell adhesion complexes and share functional similarities with nephrin. Binding of autoantibodies to these adhesion molecules results in the redistribution of desmogleins from the cell surface and subsequent loss of their expression [22].

Based on these observations, Watts and colleagues investigated the presence of circulating autoantibodies against nephrin in patients with MCD [23]. In their study, serum or plasma samples were obtained from 41 pediatric and 21 adult patients with MCD at a time when proteinuria exceeded 3 g/g creatinine [23]. An indirect enzyme-linked immunosorbent assay (ELISA) was developed using the extracellular domain of human nephrin (hNephrinG1059) as the antigen. Using this assay, anti-nephrin autoantibodies were detected above the predefined cut-off value in 18 of 62 patients (29%) [23]. Notably, among seven Asian patients included in the cohort, five were positive for anti-nephrin autoantibodies, suggesting a potentially higher prevalence in Asian patients with MCD. In addition to serological analyses, Watts et al. examined kidney biopsy specimens showing that in patients with MCD who were positive for anti-nephrin autoantibodies, conventional immunofluorescence microscopy revealed punctate IgG staining within the glomeruli [23]. Further analyses using confocal microscopy and super-resolution structured illumination microscopy (SIM) demonstrated colocalization of IgG with nephrin [23]. They also showed that serum from patients with anti-nephrin autoantibody-positive MCD immunoprecipitated recombinant nephrin as well as nephrin from human glomerular extracts [23].

Subsequently, Hengel et al. established an assay in which anti-nephrin autoantibodies were enriched by nephrin-based immunoprecipitation of patient serum, followed by ELISA quantification [24]. Using this approach, they analyzed 539 cases of idiopathic nephrotic syndrome, including pediatric patients, and reported a high prevalence of anti-nephrin autoantibodies, reaching 90% in untreated pediatric idiopathic nephrotic syndrome [24]. Horinouchi et al. evaluated circulating anti-nephrin autoantibodies by ELISA in paired plasma samples from 13 Japanese children with steroid-sensitive nephrotic syndrome obtained at disease onset and after steroid monotherapy [25]. Anti-nephrin autoantibodies were detected in 46% of patients during active disease and significantly decreased following treatment [25]. Taken together, these findings indicate that anti-nephrin autoantibodies are involved in a substantial proportion of diffuse podocytopathies in native kidneys.

## Anti-nephrin autoantibodies in post-transplant recurrent FSGS

In kidney transplantation, it is possible to perform molecular analyses using biopsy specimens obtained immediately before exposure to the recipient’s circulating blood (the 0-h biopsy) and those obtained at an extremely early time point after reperfusion (the 1-h biopsy). Following the identification of anti-nephrin autoantibodies in MCD in 2022, we examined the presence of anti-nephrin autoantibodies in a pediatric patient with post-transplant recurrent FSGS [26]. Pathological analysis of the allograft biopsy using SIM revealed IgG deposits colocalized with nephrin, which exhibited altered localization to the intracellular area of podocytes in 1-h biopsy specimens [26]. The presence of anti-nephrin autoantibodies in patient plasma was confirmed by western blot analysis [26]. Using the 1-h biopsy specimen from this case, we also reported enhanced phosphorylation of nephrin together with increased expression of Src homology and collagen homology A (ShcA), an adaptor protein that binds phosphorylated nephrin and participates in endocytosis [26]. These findings suggest that binding of anti-nephrin autoantibodies to nephrin promotes nephrin phosphorylation, leading to altered nephrin localization via phosphorylation-dependent endocytosis. This study represents the first report to demonstrate downstream molecular events triggered by anti-nephrin autoantibodies in human kidney biopsy tissue.

To clarify the positivity rate of anti-nephrin autoantibodies in post-transplant recurrent FSGS, we conducted a multicenter study of pediatric patients with post-transplant recurrent FSGS [27]. As a result, circulating anti-nephrin autoantibodies were detected in all 11 cases with post-transplant recurrent FSGS by ELISA assays, and punctate IgG deposition colocalizing with nephrin was consistently observed in allograft biopsy specimens obtained during recurrence [27]. Importantly, IgG deposition disappeared after remission in treatment responders [27]. These findings strongly suggest that anti-nephrin autoantibodies represent a key circulating permeability factor in post-transplant recurrent FSGS.

Subsequently, Batal et al. reported that pre-existing anti-nephrin autoantibodies were identified in 8 of 21 (38%) patients with post-transplant recurrent FSGS, but in none of the 17 patients without recurrence. The ELISA demonstrated 100% specificity and a 100% positive predictive value [28]. Collectively, the presence of anti-nephrin antibodies before transplantation appears to be associated with an extremely high risk of FSGS recurrence [29]. To date, de novo anti-nephrin antibody positivity developing after kidney transplantation has not been reported, and the clinical relevance of this phenomenon remains unclear.

## Pathophysiological mechanisms of anti-nephrin autoantibody-mediated proteinuria

In kidney biopsy specimens from recurrent FSGS cases with anti-nephrin autoantibodies, C3 deposition is not observed at sites of punctate IgG staining, and electron-dense deposits are not identified on electron microscopy [27]. These results indicate that immune complex formation is not involved in the pathogenesis of diffuse podocytopathies associated with anti-nephrin autoantibodies.

We also reported that exposure to anti-nephrin autoantibodies resulted in a reduction in nephrin expression from very early phases, and nephrin expression further decreased in accordance with disease progression using graft specimens obtained from patients with post-transplant recurrent FSGS [30]. In this study, we showed that Kirrel1 and CD2-associated protein (CD2AP) expression was also decreased in all anti-nephrin antibody-positive post-transplant recurrent FSGS patients. On the other hand, podocin expression did not decrease and remained localized along the capillary loops in the specimens obtained at 1 h and during recurrence in all recurrent FSGS cases with anti-nephrin autoantibodies. These findings offer new insights into the early molecular events in post-transplant recurrent FSGS driven by anti-nephrin autoantibodies [30].

Hengel et al. developed experimental immunization in mice that resulted in the development of nephrotic syndrome with an MCD-like phenotype, characterized by IgG accumulation at the podocyte slit diaphragm [24]. They performed proteomic and phosphoproteomic analyses of glomeruli and found that phosphorylation of nephrin was increased at tyrosine residue Y1191, a conserved tyrosine residue corresponding to Y1176 in human nephrin. This phosphorylation site is known for its involvement in nephrin signaling that leads to actin assembly, cytoskeletal reorganization, and nephrin endocytosis [24]. Proteomic analyses demonstrated increased expression of adaptor proteins associated with nephrin endocytosis, including ShcA [24], consistent with our observations in allograft specimens from patients with post-transplant recurrent FSGS [26, 27].

Very recently, Zhang Y, et al. reported that anti-nephrin antibodies induce phosphorylation of nephrin and ephrin-B1 in a transient receptor potential canonical 6 (TRPC6)-mediated calcium influx-dependent manner, leading to dissociation of ephrin-B1 from nephrin and Par6 using an animal model [31]. This releases Par6 to interact with and stabilize cell division cycle 42 (Cdc42), resulting in enhanced Cdc42 activity. Activated Cdc42 subsequently increases calcineurin activity suppressing nephrin and ephrin-B1 expression [31]. Collectively, these findings suggest that Cdc42 signaling represents a critical initiating pathway in anti-nephrin autoantibody-mediated podocytopathy.

## Anti-slit diaphragm autoantibodies targeting molecules other than nephrin

Recently, Raglianti et al. described anti-slit diaphragm autoantibodies—specifically anti-podocin and anti-Kirrel1 autoantibodies—in pediatric and adult diffuse podocytopathies [32, 33]. They reported IgG colocalization with nephrin, podocin, and Kirrel1 in kidney biopsy specimens from affected patients, including cases positive for both anti-nephrin and anti-podocin autoantibodies. Subsequently, we also identified one triple-positive rFSGS case using dual immunofluorescence for IgG with nephrin, podocin, and Kirrel1 [34]. In this case, dual immunofluorescence staining revealed that nephrin exhibited altered localization, shifting away from the slit diaphragm, where it colocalized with IgG, while both podocin and Kirrel1 retained their localization at the slit diaphragm and individually colocalized with IgG under SIM observations [34]. The pathogenic significance of anti-podocin and anti-Kirrel1 autoantibodies should be clarified through future studies. Habbig et al. reported that in pre- and post-transplant serum samples from a patient with FSGS, anti-nephrin autoantibodies were cross-reactive with NEPH3 (filtrin), another key slit diaphragm protein [35].

Based on these reports, examinations for autoantibodies targeting nephrin and other slit diaphragm molecules will be considered in patients with diffuse podocytopathies.

## Limitations and future directions

There is currently no established reliable method for detecting or quantifying anti-nephrin autoantibodies. To date, several assay platforms have been developed, including immunoprecipitation-based ELISA and conventional ELISA, in laboratories worldwide (Table 1) [36]. ELISA-based assays are technically straightforward, scalable, and require minimal sample volumes while enabling standardized quantification. However, in diffuse podocytopathies mediated by anti-nephrin autoantibodies, antibodies can induce profound podocyte injury despite being present at extremely low concentrations in the circulation [37]. Once the glomerular filtration barrier is disrupted, serum immunoglobulins—including pathogenic anti-nephrin autoantibodies—can traverse the damaged barrier and be excreted in the urine together with albumin and other plasma proteins [38, 39]. Consequently, circulating antibody levels may decline rapidly. As a result, conventional immunoassays such as ELISA, which rely on exceeding predefined optical density thresholds, may fail to detect autoantibodies during active disease. Therefore, serial sampling and careful consideration of disease timing are essential for accurate interpretation of serological results.

**Table 1:** Summary of detection methods and reported prevalence of anti-nephrin autoantibodies in previous studies

Study	Immunological assay for detecting circulating anti-nephrin autoantibodies	Details of immobilized recombinant nephrin	Pathological analysis	Study population and diagnosis	Race	Prevalence of anti-nephrin autoantibodies
Watts et al. 2022 J Am Soc Nephrol [8]	1. Immunoprecipitation 2. Signal-enhanced ELISA	Recombinant human nephrin ectodomain (amino acids 1–1059), derived from HEK293	SIM	41 pediatric patients with MCD 21 adult patients with MCD	Multiethnic	22% (9/41) in pediatric patients 43% (9/21) in adult patients
Shirai, et al. Kidney Int 2024 [27],	Signal-enhanced ELISA	Recombinant human nephrin ectodomain (amino acids 23–1029), derived from mouse myeloma cells	SIM	11 pediatric patients with post-transplant recurrent FSGS	Japanese	100% (11/11)
Shirai, et al. Kidney Int 2025 [40]	Signal-enhanced ELISA	Recombinant human nephrin ectodomain (amino acids 1–1055), derived from HEK 293	Not performed*	11 pediatric patients with post-transplant recurrent FSGS	Japanese	91% (10/11)
Batal et al. 2024 Kidney Int [28]	Signal-enhanced ELISA	Recombinant human nephrin ectodomain (amino acids 1–1059), derived from HEK293	SIM	38 kidney transplant patients with MCD/FSGS, of which 21 developed post-transplant recurrent MCD/FSGS	Mainly White patients	38% (8/21) in post-transplant recurrent MCD/FSGS 0% (0/17) in post-transplant non-recurrent MCD/FSGS
Hengel et al. N Engl J Med 2024 [24]	Hybrid assay of immunoprecipitation followed by ELISA	Recombinant human nephrin ectodomain (amino acids 25–1037), derived from HEK293	Not performed	182 pediatric patients with INS 105 adult patients with MCD 74 adult patients with FSGS	European cohort	52% (94/182) in pediatric patients with INS, particularly high at 90% (35/39) in patients with nephrotic-range proteinuria who had not received immunosuppressive therapy 44% (46/105) in adult patients with MCD, particularly high at 69% (24/35) in patients with nephrotic-range proteinuria who had not received immunosuppressive therapy 9% (7/74) in adult patients with FSGS
Raglianti et al. Kidney Int 2024 [33]	Standard ELISA	Recombinant protein partially covering the first IgG-like domain of human nephrin (amino acids 23–92), using commercially available ELISA kit	SIM and STED	Pathological analysis; 62 pediatric patients with MCD/FSGS 40 adult patients with MCD/FSGS ELISA; 13 pediatric patients with SSNS 12 pediatric patients with MCD/FSGS	Italian	33% (10/30) in pediatric patients with MCD 25% (8/32) in pediatric patients with FSGS 68% (13/19) in adult patients with MCD 19% (4/21) in adult patients with FSGS 38% (5/13) pediatric patients with SSNS 33% (4/12) pediatric patients with MCD/FSGS

*ELISA* enzyme-linked immunosorbent assay, *HEK* human embryonic kidney, *SIM* structured illumination microscopy, *STED* stimulated emission depletion microscopy, *MCD* minimal change disease, *FSGS* focal segmental glomerulosclerosis

*Same patient cohort as reported by Shirai et al. in Kidey Int 2024[27]

Assay specificity may also be influenced by the choice of recombinant nephrin, as proteins expressed in non-human systems may differ in post-translational modifications and potentially generate false-positive signals [40]. Moreover, similar methodological challenges apply to assays designed to detect autoantibodies targeting slit diaphragm molecules other than nephrin.

In addition, although evidence supporting the pathogenic role of anti-nephrin autoantibodies is increasing, the functional significance of autoantibodies directed against other slit diaphragm molecules remains unclear. Further investigation is required to elucidate their mechanistic roles and contributions to the pathogenesis of diffuse podocytopathies.

Strategies aimed at preventing post-transplant FSGS recurrence remain incompletely established, and the role of pre-transplant rituximab administration and plasma exchange remains controversial [41]. Additionally, it remains to be determined whether anti-nephrin autoantibody titers can serve as predictive biomarkers for post-transplant recurrence. Habbig et al. provided important insights through a case report demonstrating a personalized, prospective monitoring strategy that evaluated responses to individual therapeutic plasma exchange sessions and incorporated close surveillance after kidney transplantation [35]. Further studies are urgently required to establish effective preventive strategies.

## Conclusion

The discovery of anti-nephrin autoantibodies represents a paradigm shift in the understanding of diffuse podocytopathies. Elucidating the mechanisms of autoantibody-mediated podocytopathy and developing targeted therapies based on this knowledge are urgent and promising directions for future research.
